# “Right now we are scared of each other, we fear everyone, the whole world has COVID”: The impact of COVID-19 on young female sex workers in Kampala, Uganda, during national lockdowns in 2020–2021

**DOI:** 10.1371/journal.pgph.0001268

**Published:** 2023-01-05

**Authors:** Rachel King, Ritah Namugumya, Catherine Namuddu, Femke Bannink Mbazzi, Francis Xavier Kasujja, Judith Nankabirwa, Janet Seeley

**Affiliations:** 1 Institute for Global Health Sciences, Department of Epidemiology and Biostatistics, University of California, San Francisco, (UCSF), San Francisco, California, United States of America; 2 Medical Research Council/Uganda Virus Research Institute and London School of Hygiene & Tropical Medicine (MRC/UVRI & LSHTM) Uganda Research Unit, Entebbe, Uganda; 3 Department of Global Health and Development, London School of Hygiene & Tropical Medicine, London, United Kingdom; Institute of Development Studies, UNITED KINGDOM

## Abstract

In 2020–2021 the COVID-19 pandemic led to multiple and diverse global public health response strategies globally and in Uganda to slow the spread of the virus by promoting wearing face coverings in public, frequent hand washing, physical distancing, restricting travel, and imposing home lockdowns. We conducted 146 interviews over four rounds of phone-follow up calls over 15 months with 125 young female sex workers coinciding in time with four different government-imposed lockdown periods in Kampala, Uganda, to assess the impact of these measures on young sex workers, their families and their communities as well as to gauge their resilience. Our findings revealed how COVID-19 fears and public health restrictions over time pushed an already marginalized population to the brink and how that pressure drove some participants into a new way of life.

## Introduction

Globally, by mid-2022, over 500 million people had been infected with COVID-19, with numbers increasing on a daily basis [1]. In all African countries, over 8.2 million confirmed COVID-19 cases have been reported with over 170,000 deaths [2]. In Uganda, 163,944 cases and 3,500 deaths have been recorded [3]. In many low-income communities around the world, the poorest, most congested neighborhoods lack access to basic water, sanitation and hygiene to protect themselves from the virus.

Since the reporting of confirmed cases of COVID-19 on March 21^st^, 2020, the Ugandan Government quickly implemented a public health response. The measures put in place were based on the WHO recommendations, adapted specifically to Uganda, and included promoting wearing masks, regular hand washing and social distancing; closure of borders, schools, and religious institutions; and recommendation or mandate to work from home for all individuals in non-essential occupations. Since March 31^st^ 2020 a ban on all private and public transport lifted after three months and reinstated for two months in June and July 2021, and curfew from 7.00pm (changed to 9pm in June 2020) until 6.30am initially for a period of 14 days which was extended continuously for a total of almost 2 full years [4].

Research on COVID-19 has been conducted in many countries hit by the pandemic, often focusing on the epidemiology of the disease, its medical determinants, treatment and vaccines [5]. Early studies described the economic, social, and psychological effects of the COVID-19 measures in high and middle-income countries [6–9], as well as the anxiety around the virus and associated illness and death [10, 11]. Health workers in Zimbabwe and South Africa noted how implementation of prevention measures was difficult for people without access to water, food security, and a daily income [12, 13]. Research in Uganda has so far reported how health workers had sufficient knowledge and beneficial attitudes but poor practices related to COVID-19 [14]. Further, studies in Uganda, on COVID-19 have reported how the COVID-related public health restrictions affected people living in informal lower income settings as well as adolescent boys and young men through increased mental health challenges, poor access to food and a reduction in income [15, 16]. Studies in the country have also emphasized increased rates of child abuse and intimate partner violence as well as violence in general during lockdowns [17–19].

Globally, before the availability of vaccines, the COVID-19 mitigation strategies have focused on limiting virus spread by promoting and enforcing the key infection prevention and control public health messages and measures mentioned above. Treatment was largely supportive and topics that had been relevant to the different stages of the COVID-19 pandemic include: perceptions of risk or threat, views on leadership, public trust or mistrust in political and health leadership, individual and collective thinking related to vaccine uptake as well as protective measures, science communication (fake vs real news, persuasion and conspiracy theories), social context (social inequality, social norms), stress and coping (social isolation, connection, economic stress) [20]. These topics become even more important for already marginalized communities.

Some recent studies on vulnerable populations such as sex workers in low- and middle-income countries have reported on this population’s access to health care during COVID-19 [21–24]. Globally, vulnerable populations are often marginalized, economically disempowered, experience poor social conditions (violence, poor housing, food insecurity, etc.), lack access to health care, education, social services and may practice sex work to survive even though the profession is illegal in many countries. A pervasive combined stigma has been reported in relation to COVID-19 and HIV as some patients have been struggling to access routine drug refills for family planning, anti-retroviral therapy (ART) and pre-exposure prophylaxis (PrEP), especially if they have not disclosed their HIV serostatus through their networks or to close family members [23].

Young women aged 15 to 24 years who engage in high-risk sexual behavior (YWHR) are one of the most-at-risk populations for HIV in sub-Saharan Africa [25–28]. In Uganda, HIV prevalence among this group has been estimated at 26% compared to a national prevalence of 4.9% in the general population same age group. Moreover, young HIV-uninfected women are particularly vulnerable to numerous health-related challenges such as sexually transmitted infections (STI) including HIV intimate partner violence and unplanned pregnancies [25]. Young women who engage in transactional sex are especially susceptible to HIV due to male partner dominance, the difficulty of negotiating safe sex with clients, stigma and discrimination [25, 26]. Violence or the fear of violence can make it further challenging for women to ensure safer sex and to use and benefit from health services [25, 29–31]. During the global COVID-19 pandemic, this population was critically vulnerable due to these and a myriad of layered factors.

Additionally, sex work is associated with mobility [32, 33]. Mobility can place people in situations that increase their risk of poor health outcomes [32, 34–36]. Irregular housing status, language and cultural barriers, charges attached to services, lack of youth-inclusive health policies and prevent inaccessible services mobile people from accessing the health services they need [33].

As in other places, the COVID-19 public health measures that were put in place in Uganda particularly affected small businesses that were struggling to survive. Although limited data are available for Uganda, available information showed that vulnerable populations, including impoverished rural and urban households and sex workers (some of whom were living with HIV and on ART) who were already struggling to feed themselves and their children, faced even more difficulty after their daily earnings either reduced or disappeared completely [37, 38]. Some of the COVID restrictions related to physical movement and lockdown pushed these already vulnerable populations to the brink of desperation [39]. A randomized survey of 1277 households conducted between March and May of 2020 noted that households in Uganda purchased 50% less food and were 50% more likely to miss a meal and a survey in Kampala found that households in the lowest income bracket were the hardest hit [38].

The present study is a sub-study of the ZETRA study, a randomized controlled trial implemented from November 2016 to the end of January 2022 in Kampala, Uganda. ZETRA enrolled 644 participants who were 15–24 years-old, HIV-negative and engaged in sex work. Participants were randomized to either a behavioral intervention (seven sessions; 4 group and 3 one-on-one, led by a trained counselor) covering topics including health literacy and social media skills development, or control arm that received standard of care at specialized clinic for high-risk women in Kampala. All participants were invited to attend study visits at months 0, 6, 12 and 18 post-enrolment; they used Google maps software to identify work venues at 12 and 18-month visits to assess mobility. By locating their work venues at two time points, participants could be seen to have moved to different places over a six month period [40].

This sub-study aimed to understand how the current COVID-19 pandemic and associated restrictions impacted the daily lives of these young sex workers to inform interventions. We assessed: participants’ knowledge of COVID-19, the Ugandan government’s related public health response as well as how each phase of the response impacted this highly vulnerable population, their families, and community members’ daily lives including health, health care access, children’s education, economic potential, and their social and psychological wellbeing. With an understanding of this populations’ range of knowledge, range of impacts on health and well-being, interventions can be better tailored to impact the population they serve.

## Methods

**Fig 1:**
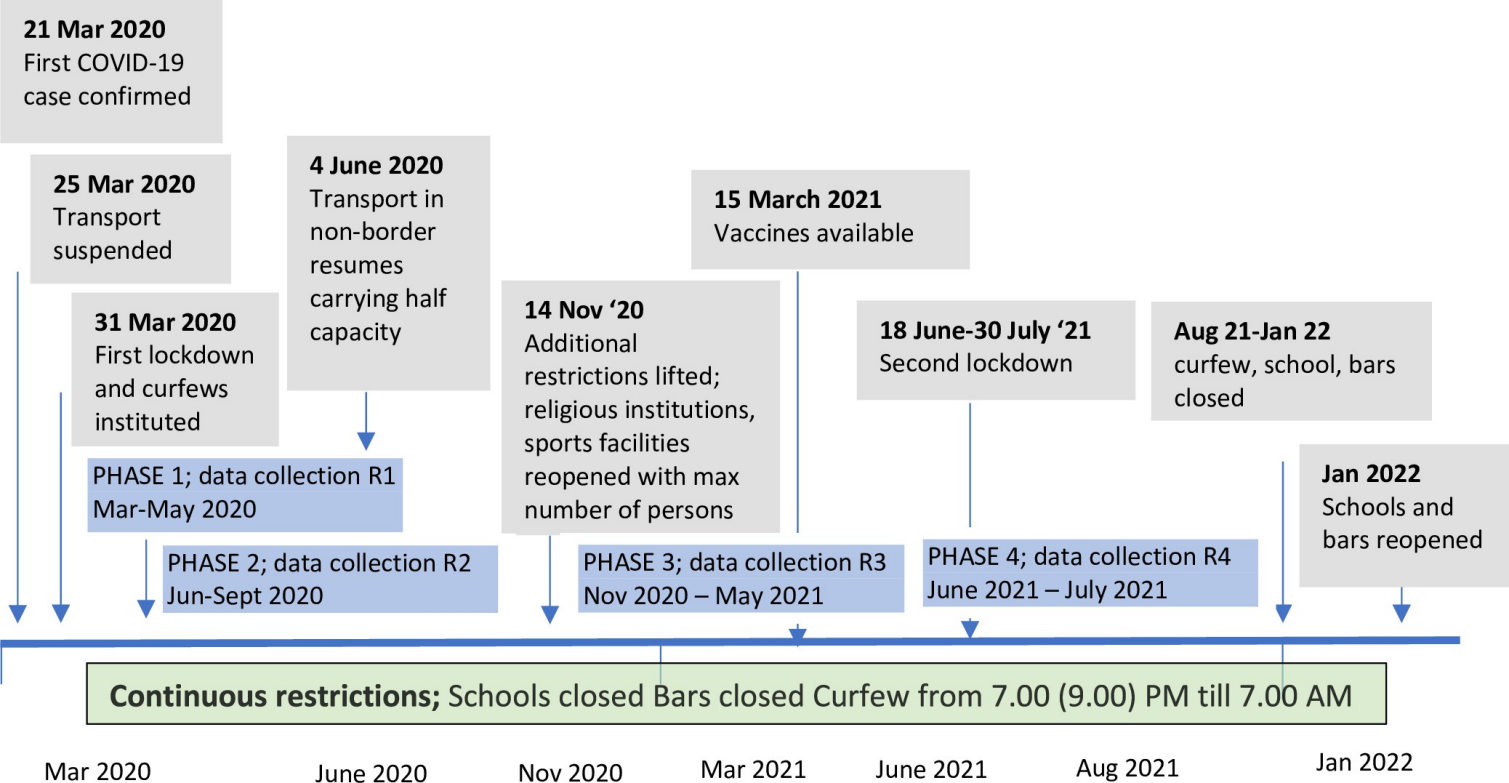
Summary of COVID national public health restrictions and data collection rounds; May 2020-January 2022.

All ZETRA study participants who were still in under follow-up as of May 2020 were eligible for this sub-study. The study consisted in phone follow-up interviews at four time points that corresponded to the different national COVID-related restriction phases (Fig 1). Due to submission of IRB and subsequent approval, data collection in round one started in May, while the COVID restrictions started on 31 March 2020. Due to participant mobility and phone contacts, it was not always possible to contact every participant in all rounds of the survey. Some participants traveled out of the study area and may not have been able to answer phone calls.

The phone follow-up was conceived of as a rapid qualitative sub-study in the form of serial individual interview design. The interviews lasted 15 to 30 minutes and included open-ended questions around general knowledge about COVID-19, the government’s public health response, as well as beliefs, perceptions and experiences on how COVID-19 had been impacting their own lives, and their families and communities.

Interviewers were counsellors and mobilizers of the ZETRA study who had known study participants for at least 24 months and were trained on the study protocol. They initiated the phone calls by carefully introducing the study as a follow-up on the ZETRA study participants considering the impact of COVID-19. To mitigate the risk of people eavesdropping or participant’s being uncomfortable talking on the phone with household members present, counsellors specifically asked the participants if they would like to speak on phone in a separate room and/or outside to allow them some privacy, or at a different time. Participants were given the opportunity to ask questions at any time during the interview, and staff members were trained on how to answer basic questions and, when necessary, to refer to a relevant health facility. If consented, the phone call was recorded by the study team member conducting the interview to ease data transcription. When recording was not consented, phone calls were not recorded.

### Data storage

Data storage followed GDPR guidelines; no personal identifiers were included on any recorded or transcribed documents. Computers and phones were all password protected. All recordings were deleted after transcription.

### Data management and analysis

**Fig 2:**
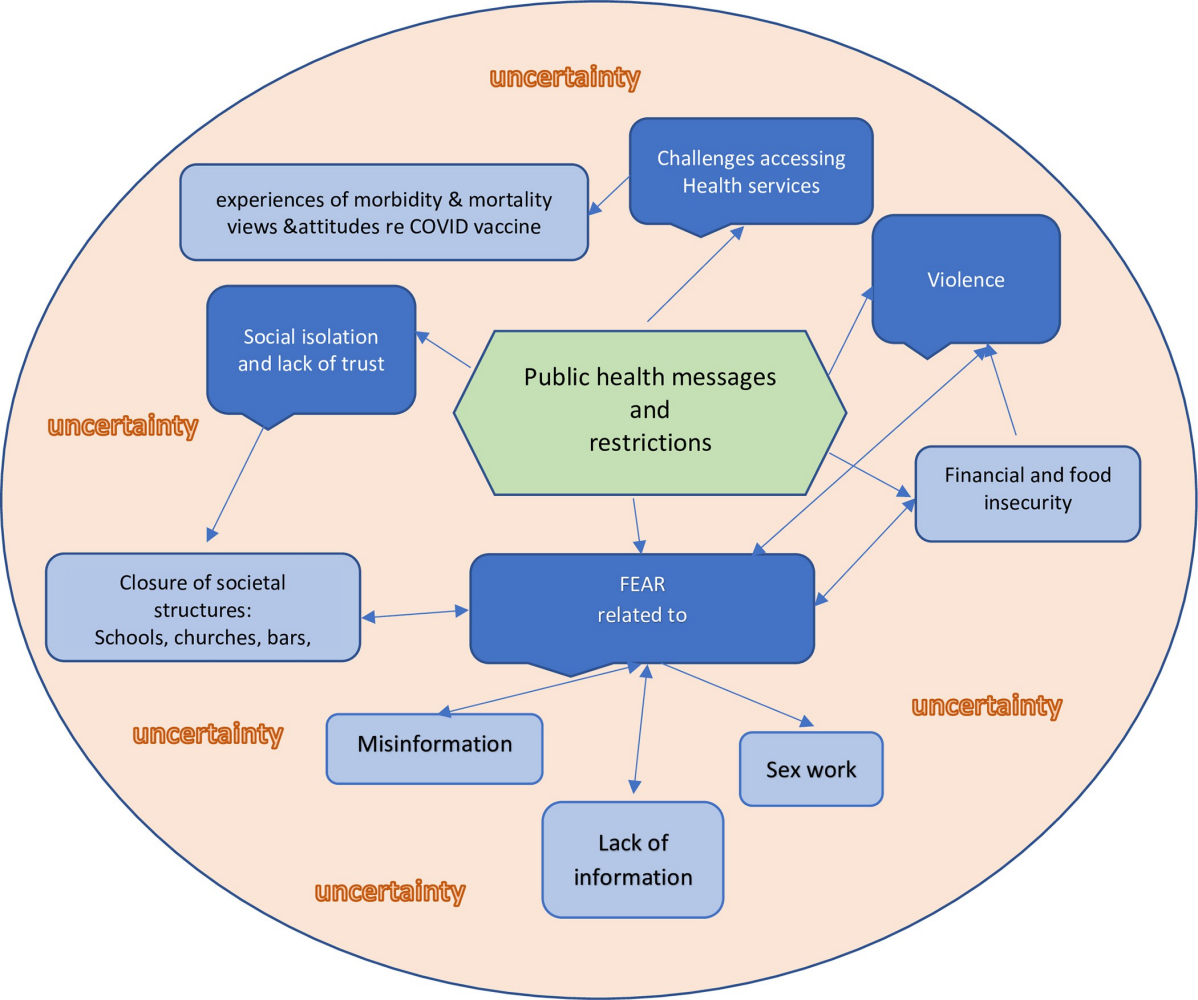
Themes related to public health messages and restrictions; Kampala 2020–2022.

The data were transcribed and translated into English. Data were managed using NVivo for Mac 11.1.1 and analyzed using framework thematic analysis and Interpretive Description [41, 42]. Analysis was conducted by the PI and checked with the interview team for validity. Analysis guidelines included three distinct stages: theme development, theme validation and code use, which were used as the primary analytic strategy for thematic coding with an emphasis on descriptive vs interpretive themes [43]. After reading two transcripts, a codebook of themes was developed based on the interview topics as well as those emerging from the data. Two more transcripts were then reviewed to include additional topic areas and themes. This process was repeated until a sample of 12 transcripts had been reviewed and the codebook reached a stage where no new themes or topic areas emerged. All transcripts were then coded using the final version of the codebook and merged using NVivo for Mac 11.1.1 software before themes were summarized across respondents. Analysis focused on identifying the dominant and the range of explanations and comparisons across clients stratified by round number. We tried to link responses to levels of restriction in order to inform a potential public health response for particularly vulnerable populations (Figs 1 and 2). The theoretical foundation to interpret this data used constructs from practical social justice and ecological interpretation of resilience [44].

**Fig 3:**
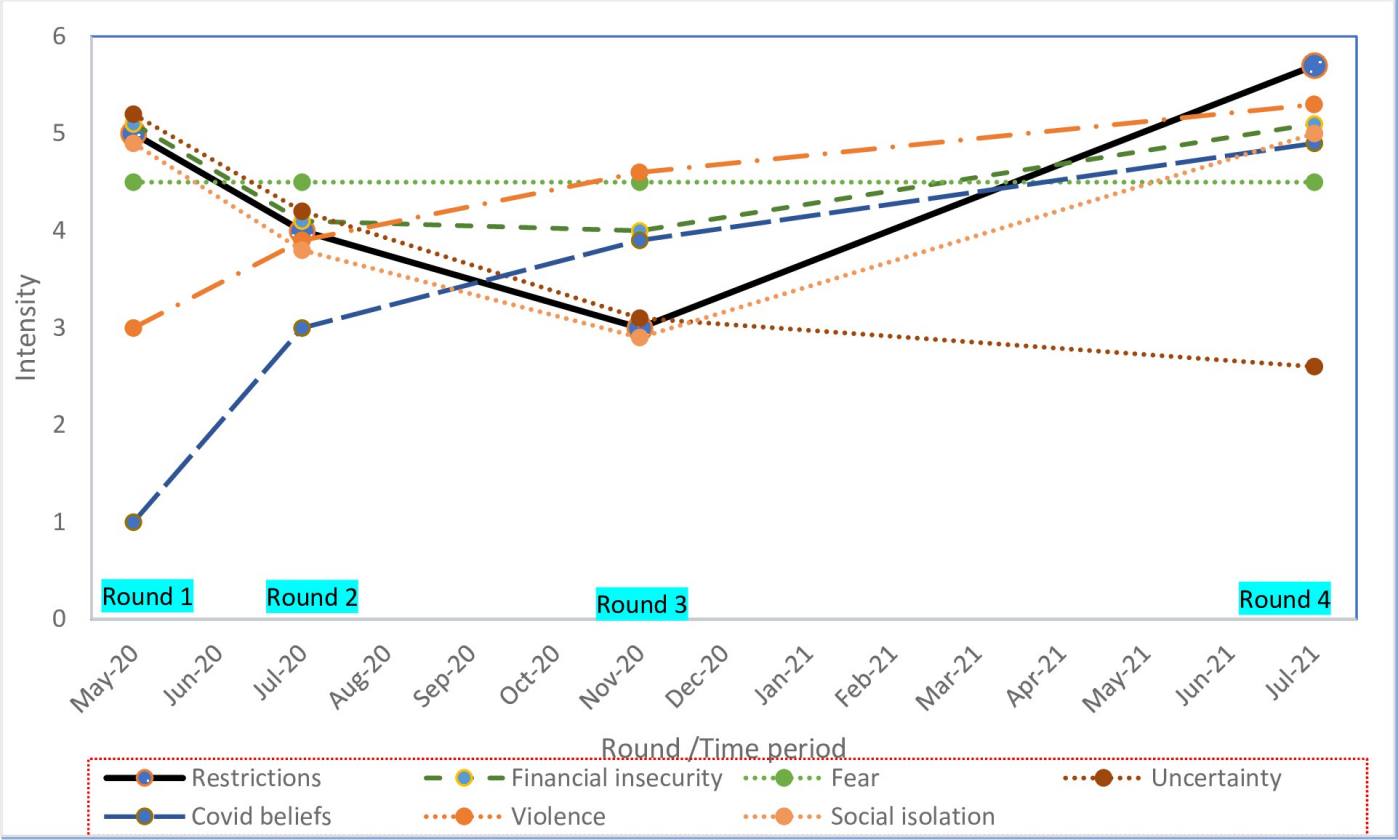
Intensity of themes over time and data collection rounds 1, 2, 3, 4 (intensity defined as a combination of frequency mentioned and words used to describe strength of feelings).

A secondary focus of the data analysis was to document and interpret changes in participant’s experience over time. To visualize these changes in parallel with changes in restrictions, we plotted the main themes across the four data collection rounds and the different government public health restrictions (Fig 3).

Interactive discussions were held with the analysis team and senior researchers to validate data interpretations and resolve any interpretation discrepancies. Preliminary findings were discussed with a group of participants to assess validity. Eight participants were selected who were still enrolled in the parent study and able to return to the clinic on 19 February 2021. Counsellors presented preliminary results to these participants, discussed each of the topics to assess whether they felt that the analysis was valid or not.

### Ethics

The ethical issues for this study were complex [45]. The study staff members had to navigate participants’ expectations, as well as sensitivity and security issues. At the start of the phone call, participants were requested to verbally consent to answering questions about how they were doing generally and additionally about COVID-19. This verbal consent was recorded by the person conducting the phone interview; the consent time and date, and the person taking the consent was recorded in a notebook. Presenting the study in order not to raise expectations was critical. Some of the data on livelihoods during the time of total lockdown was quite sensitive as bars and lodges were banned. To protect our study participants, we did not link any personal identifier data to any particular participant. We clearly explained procedures for keeping the information shared on the telephone secure, and asked participants to conduct the telephone interview in a private space. All staff received training on managing the provision of advice and information on referral pathways whenever required.

Ethical clearance was sought from the Uganda Virus Research Institute Research Ethics Committee, and the London School of Hygiene & Tropical Medicine Ethics Committee. Research clearance was obtained from the Uganda National Council for Science and Technology. For the phone interviews specific to this sub-study, verbal informed consent was obtained. Verbal consent was formally requested over the telephone and documented. Participants were compensated for their time (approximately $2.50 USD per interview) using mobile money through their phones sent after the interview.

## Results

We conducted 146 interviews over a period of 15 months of 125 participants as some participants were interviewed multiple times (7 participants were interviewed 3 times and 14 participants were interviewed 2 times). The mean age of participants was 21 years old with about nine percent having completed secondary school (Table 1).

**Table 1:** Demographic characteristics of telephone interview respondents (n = 125 participants).

Interview Round	Total number of interviews	Age (Mean)	Completed Secondary Level of education, n
1	32	20	3
2	61	18	3
3	13	21	3
4	40	21	3

Participants were asked what information they understood regarding COVID-19, where they accessed the information and how the responded to the information. Everyone (n = 125) had already heard of COVID-19 even in round one, mostly through radio, with some through television, the public address system and neighbors. Participants generally mentioned that they followed government recommendations by washing hands and staying at home.

In addition to asking about basic COVID-19 information in round one, themes that emerged in the four rounds of data collection included four main themes: i) many aspects surrounding fear, ii) social isolation, iii) violence, difficulties in accessing health services. Six main sub-themes that emerged included: experiences and views on information and misinformation, specific sex work related fear, financial and food insecurity, the effects of closure of societal structures such as schools, churches, bars and nightclubs and experiences of morbidity and mortality as well as participant views and attitudes about the COVID vaccine (especially in the last round) all embedded in the pervasive fog of uncertainty (Fig 2).

### I. Layered dimensions of fear

Almost all participants across all four rounds of data collection described different types and layers of fear (Fig 2). Despite most of our participants mentioning correct basic information about COVID-19, fear was related to the uncertainty of how this pandemic would end (particularly in round 1) and the novelty of this disease that was being defined and described by the outside world. Fear was also generated by the closure of social structures such as places of worship and the violence perpetrated by local defense units who were enforcing restrictions including curfew or gathering. The main source of fear, however, came from financial and food insecurity as the opportunities for making an income had been completely curtailed by the curfew and closure of bars and lodges. Almost all participants were completely out of work in rounds one and four, while some mentioned the ability to eke out a meager income in rounds two and three when some of the restrictions such as the transport ban were partially lifted. In general, in round one participants were really scared of COVID-19 and immediately stopped working, however in rounds two and three, some found ways to work to survive by washing and/or sanitizing their whole bodies and those of customers and wearing masks. …*Sometimes when you get a client for sex work*, *it’s very hard to refuse even if we are aware that COVID is there*, *we just go because if you refuse*, *you die of hunger even before you remember that COVID kills (22 years old Round 4*).

Many women respondents repeated that the situation was extremely bad for them especially with regards to being able to feed themselves and their children. The 7pm-6:30am curfew also limited their income because it corresponded to the time period when most participants were able to work. Public transport was completely banned during the 10-week total lockdown between 25 March and 4 June 2020 and again during the second lockdown between 18 June and 30 July of 2021, so many customers could not reach them or access their places of work.

Bars were closed during the entire duration of the study where most of our participants worked, and their customers did not have the extra money to spend on purchasing their services. Women reasoned that, as sex work was not an essential service, they had to stop work. *The only thing that worries me is money*… *generally*, *work*. *You wake up in the morning when you don’t have money and then you start worrying about what the child is going to eat*. (20 year old; Round 1)

The consequences of no income, and therefore inability to pay rent, in some cases forced participants to flee to rural areas and abandon their Kampala lives completely. Though some restrictions were lifted in rounds two and three, the curfew was enforced continuously from March 2020 to January 2022 throughout Uganda with small changes in time, (from 7pm to 9pm in rounds two and three), which had a strong impact on participants. Thus, lack of food was still apparent in round two and three even though some of the restrictions were lifted. *We don’t have anything to eat*. *Water enters our houses*. *15 people in the home*. *(18 year old; Round 2)*

By Round four, some participants were pushed to the brink of desperation.

*Things have changed a lot*, *you can see we are now back in the lockdown like we were previously*, *no moving*, *no seeing our clients*, *we are just here*! *This time the situation is worse compared to the first [lockdown] because we had just come out of a lockdown*… *Some [of us] had spent all their saving and after lockdown we used to survive on daily earnings*. *We had not yet stabilized to save money for another lockdown that is why the situation is worse*. *(26 year old; Round 4)*

### II. Social Isolation and lack of trust

During the complete lockdowns in rounds one and four, participants stressed how staying at home without interacting with others had a strong influence on children; and the silent spread of the virus instilled a lack of trust in others.

This lack of trust was highlighted particularly in relation to sex work as participants could not maintain their work when they could not trust that potential clients were not infected with the COVID virus. In round one, participants who had travelled to border areas expressed that their fear was much greater there as Uganda’s COVID-19 hotspots were mainly around the borders with Kenya, Tanzania and South Sudan during that time. Though this picture changed rapidly in following months, the stigma around sex work, truck drivers and foreigners at that time was reminiscent of the days of the HIV pandemic in the late1980s.

*My worries are many*, *Musawo [Doctor]*, *because if am walking and I get a deal [customer]*, *I worry for my health because I think*: *what if this person has Corona and I don’t know*, *what will happen to my life*? *I even fear getting close to my friends*…, *we don’t know who has and who does not have Corona; otherwise*, *we are just praying to God*. *(20 year old*, *Round 1)*

In all four rounds, many participants claimed they were depressed, they didn’t have friends or family members to share their thoughts of feeling helpless which caused anxiety and stress. The constant pressure of the children they had to take care of, added to feelings of loneliness and stress. In addition, social isolation was particularly relevant in relation to the closure of social structures like schools, churches, and not being able to attend important social and cultural functions such as burials, impacted everyone here as it did globally. Many participants were concerned about their children’s education. Some classes were taught on television; yet for many, this was inaccessible as many did not own a TV. Some had just finished paying school fees when the schools closed. One participant explained that even when schools re-open, they would not be able to afford the fees. As school closures lasted for two full academic years in Uganda, the data differed in each round with respect to the actual effects of school closure. In round one, participants did not know what to expect and in round four, the consequences of missing two years of school were very evident.

…*it pains me because my child was just going to start school and that is when they closed the schools*. *(20 year old; Round 1)*

*It has really affected both the parents and grownup children because most girls in this community are pregnant now*, *they are now sexually active*, *you find a child of 10 years pregnant*. *Parents have tried to protect the children but the children have spent time without going to school*. *The more she stays at home*, *the higher chances of falling victim to such situations*. *Even us grownups got tired of sitting in one place; how about the children*? *(20 year old*, *Round 4)*

In the middle two data collection rounds, participants felt that there was still hope that their children would be about to go back to school after one missed year and repeat that year only.

### III. Violence

Some participants explained how the desperation, uncertainty, and lack of income had stressed them and family members to a point where there were heightened tensions at home, sometimes leading to violence.

*Yes*, *you know I got a new boyfriend and I have been staying at his home but the biggest challenge with him is that when he doesn’t get money*, *he transfers the anger to me and beats me up*. *(22 year old; Round 3)*

Participants also noted how curfew and social distancing were being enforced with arrests and violence by police and local defense units. There were police roadblocks all over Kampala and along main roads outside of Kampala.

… *the problem with curfew is the security forces beating people to go home*, *they are not supposed to force adults like children to go back home; he or she knows that they have to go home*. *Most people work at night*, *so when you combine lockdown and curfew*, *it is like telling people to stay at home and sit down yet we have to survive*, *and the government has not provided any support*. *(24 year old*, *Round 4)*

Some also mentioned the feeling of lack of freedom and many young women felt forced to get married in desperation of being taken care of. Some participants talked about getting married for the wrong reasons which could result in violence when marital expectations were not met.

### IV. Challenges accessing health services

The concerns raised with regards to accessing medical care centered around closure of facilities, lack of transport (for both service providers and patients), and lack of money to pay for services or prescriptions. *In this current situation we no longer have anywhere to get condoms*. *(21 year old; Round 1)*

Many participants noted that they had no access to medical services; some mentioned specific conditions such as pregnancy or sexually transmitted infections. One participant discussed how she has started self-treatment with herbal medicine.

*I was worried about family planning*. *I was worried that it may expire and yet I can get a call from a customer anytime*. *So*, *I was scared that I may get pregnant when [my] family planning [drugs] expired*. *(21 year old; Round 2)*

A few participants noted that condoms and family planning were available at nearby health facilities, but many reported that they no longer had access to either of these services during the periods of total lockdown. Access to health services was challenging particularly during rounds one and four because of complete lockdown and a ban on public and private transport which made it difficult for both patients and health care providers to reach health facilities. Some smaller facilities were completely closed during the first lockdown or were unable to handle issues not related to COVID-19; one participant mentioned she could not get treatment for a sexually transmitted infection. In round four, participants were afraid to go to health facilities as they did not want exposure to COVID-19.

*Right now*, *we fear going to health facilities because there is COVID*, *and we might get it from there; besides most people are now looking at COVID-19 as a priority and not these other things like family planning*. *(24 years old; Round 4)*

In the first three data collection rounds, the fear of catching the virus was less acute.

### V. Changes over time

Participants mentioned many different aspects of how Corona virus and prevention measures had been impacting their lives and their family members’ lives to varying degrees or intensity over time (Fig 3).

To assess how each of the changes in public health restrictions impacted our population group, we plotted an estimate of intensity, defined as a combination of frequency mentioned and strength of feelings expressed. As seen in the figure, all themes increased over each phase and data collection round except for uncertainty which decreased which we speculate was related to more information available over time. Round one characterized by full lockdown showed high levels of uncertainty, social isolation, fear and financial insecurity. Feelings of uncertainty reportedly decreased as more information on COVID-19 became available over time through newspapers, national public address by health workers and the president. In rounds two and three, when restrictions were partially lifted, participants seemed to have hope that life was going to get back to some semblance of normal, even if schools were not yet opened and curfew was still in place; this was reported with small decreases in levels of financial insecurity and social isolation. Some participants mentioned in rounds two and three how they figured out how to make some money by seeing a few customers during the day. Other income-generating activities that participants mentioned included making and selling masks, teaching nursery children since schools were closed, and travelling to rural areas to start agriculture. The changing of curfew from 7pm to 9pm in phase 2 of government COVID-19 restrictions helped some businesses to improve slightly.

*We are poor; we don’t have food*. *The lockdown was relaxed however the customer who used to give you 10000/ = [Ugandan Shillings (UGX)] will tell you I’m broke*. *He will tell you to wait until business is good*, *and on top of that there are riots [due to presidential elections]*. *A guy can give you 1000/ = [UGX] and you want to refuse yet you too have to earn a living (24 year-old; Round 3)*

There were, however, reports of COVID-fatigue, which was different from Rounds 1 and 4.

*People no longer care whether COVID-19 exists or not*, *they want all things to go back to normal and they do their work freely*. *They say that let all business operate like before*, *those who die will die and those who survive will survive*. *(26 year old; Round 3)*

In round four (June-August 2021), many participants had witnessed severe illness and many deaths. The previously expressed weariness regarding restrictions, seemed to be replaced by beliefs in the severity of the pandemic.

*What I know about the current COVID 19 wave is [it is] so dangerous because we now see the patients and those who die of COVID 19*, *it is not like the previous wave where we used not to see the patients*. *(20 year old*; *Round 4)*

In addition, participants were asked about their views on the COVID-19 vaccine. As in other contexts, participants had heard of the vaccine, and many were anxious to get their dose. Some however reported fear of the vaccine and cited misinformation campaigns as some of the reasons.

*Doctor*, *I was afraid of it*. *Do you remember when files were taken to the radio station*, *and it was broadcast that 800 people had been given the wrong vaccine*? *It was broadcast over the radio*. *Many people had been vaccinated that time*. *(22 year old Round 4)*

By round four of data collection, many participants demonstrated an ability to learn from their experience especially in round 1 due to the total lockdown. This was also evident in rounds two and three, pointing to the development of a resilience that helped carry participants through the months before restrictions were lifted.

## Discussion

Our findings show and agree with similar studies in sub-Saharan Africa that initial and ongoing COVID-19 public health prevention measures can have deleterious impacts on already vulnerable populations of young sex workers [46, 47]. We assessed emerging themes in relation to historical public reactions to past pandemics. As other studies in sub-Saharan African have found, despite reports that participants understood and believed the public health messages and the importance of standard operating procedures, implementing them was not only difficult, but in some cases impossible, due to structural constraints and the crowded and insecure way young sex workers live, their lack of financial resources and loss of income to buy basic necessities, and their obligation to leave home to make enough money on a daily basis [13]. Many young sex workers in Kampala live in crowded conditions with no access to running water or disposable income [16].

We investigated participants’ ability to access health services during COVID-19 restrictions and found, as in other countries, that during lockdowns and when public and private transport was banned, study participants had serious difficulty accessing any health services [48]. This impacted particularly severely young urban sex workers as they are particularly at risk for unintended pregnancy and sexually transmitted infections. Lack of both preventive and care services leaves the population at risk of preventable illnesses, and highly vulnerable when unable to access critical care [48]. As others have reported in Uganda, the already stretched health care system focused its energy on assessing and addressing COVID-19 challenges which meant that all other health challenges such as family planning and condom distribution were left with many fewer resources showing that restrictions, especially in movement can lead to increased deaths among the most vulnerable [48]. In addition, with no readily available cash, our participants were unable to access facilities that were open during the lockdowns when some smaller facilities had to close.

Our study, as others, have highlighted that the COVID-19 pandemic and associated restrictions have had vastly unbalanced impacts on different populations and have exacerbated many pre-existing vulnerabilities across socio-economic lines as well as class and gender [49]. The lockdowns and the protracted length of curfew and school and bar closures in Uganda impacted young sex workers across all domains of their lives from the personal, emotional to the structural and relational. These impacts, though felt across the globe, have had a greater impact on people whose daily survival depends on the ability to move around the city, to have close contact with customers and use night time hours to work and survive [22, 39]. This pandemic appears to have shed light on some of the hidden inequities, for example where those with money can access schooling online or on television and those without, cannot. Our findings highlighted how the inability to continue their work life, led participants to feelings of fear and isolation, increased poverty and violence.

Looking through the lens of history has highlighted that topics such as: threat perception, leadership, science communication, social context, stress and coping help frame responses to a new pandemic [20]. In particular, how individuals perceive the risk of catching an infectious agent, whether they panic or ignore the risk perhaps because of other more urgent priorities, especially when it is not apparent in daily lives; how individuals feel about the leadership, the political leaders and the health leaders. In its response, Uganda has used both social leaders in its campaign, the President himself in regular national addresses throughout the pandemic, as well as the health ministers and local celebrities [50]. This has proven successful in promoting basic health information on COVID-19 prevention which was apparent already in May of 2020. Some participants cited learning COVID-19 information from the regular presidential address.

Our findings highlight that interventions in a context of an emerging pandemic must consider populations that live on a daily wage, persons that cannot implement all the SOPs dictated by government, and populations that depend on moving around the city to survive. If public health measures are to be put in place to protect the population during a health crisis, national public health programs must find ways to deliver services to populations most at risk and most in need in a way that averts or limits the negative impact of public health restrictions for the most vulnerable. Specific intervention strategies must be developed and tailored to these populations and implemented in a way that ensures their well-being, even during a pandemic.
